# Correlation between physical anomaly and behavioral abnormalities in Down syndrome

**DOI:** 10.4103/1817-1745.76096

**Published:** 2010

**Authors:** Ranjan Bhattacharyya, Debasish Sanyal, Krishna Roy, Sumita Bhattacharyya

**Affiliations:** Department of Psychiatry, Calcutta National Medical College, Kolkata, India; 1 Department of Paediatrics, Nil Ratan Sirkar Medical College, Kolkata, India

**Keywords:** Behavioral abnormalities, correlation, Down syndrome, minor physical anomaly

## Abstract

**Objective:**

The minor physical anomaly (MPA) is believed to reflect abnormal development of the CNS. The aim is to find incidence of MPA and its behavioral correlates in Down syndrome and to compare these findings with the other causes of intellectual disability and normal population.

**Materials and Methods:**

One-hundred and forty intellectually disabled people attending a tertiary care set-up and from various NGOs are included in the study. The age-matched group from normal population was also studied for comparison. MPA are assessed by using Modified Waldrop scale and behavioral abnormality by Diagnostic assessment scale for severely handicapped (DASH II scale).

**Results:**

The Down syndrome group had significantly more MPA than other two groups and most of the MPA is situated in the global head region. There is strong correlation (*P* < 0.001) between the various grouped items of Modified Waldrop scale. Depression subscale is correlated with anomalies in the hands (*P* < 0.001), feet and Waldrop total items (*P* < 0.005). Mania item of DASH II scale is related with anomalies around the eyes (*P* < 0.001). Self-injurious behavior and total Waldrop score is negatively correlated with global head.

**Conclusion:**

Down syndrome group has significantly more MPA and a pattern of correlation between MPA and behavioral abnormalities exists which necessitates a large-scale study.

## Introduction

Minor physical anomalies (MPA) are defined as the unusual morphological features that are found in less than 4% of the general population.[1] The relationship between physical characteristics and behavior has intrigued man for centuries. Galen, a Roman physician and major medical authority during the Middle Ages, began the physiognomy era and advocated the view that physical features could reflect inner characteristics of behavior.[2] The concept of physiognomy suggest that deviant behavior could be predicted from certain physical characteristics of the head and hands.

The specific physical deviations, such as jaw size and facial asymmetries can be related with the tendency to turn to criminal behavior.[3]

The MPA do not directly cause behavioral deviances rather serve as markers for some fetal disturbance of development in the first and early second trimester.[4]

The children with oral disruptions can have difficulty with socializing and may have neurological deficits from feeding difficulties during the first few months of life.[5] It is, therefore, hypothesized that disruptions during a critical stage of development of a physical feature can cause MPA that can lead to a change in brain development that can cause other behavioral problems. The relationship between MPA and behaviors is considerably consistent in males than in females. Furthermore, there is also a relationship between MPA and obstetrical complication.[6]

In the absence of an identifiable syndrome, an increase in MPA have been reported in several groups including newborns,[7,8] school-aged children,[9] schizophrenic and autistic youngsters,[10] intellectually disabled children,[11,12] psychoneurotic children, learning-disabled children,[13,14] speech and language-impaired children,[1] hyperactive children[1,14] and inhibited children.[8] MPA have major informational value for diagnostic, prognostic and epidemiological purposes. They provide an important clue to specific malformation diagnosis, brain pathology and timing of pathology.[15,16] A study by Steg and Rapoport reported a mean Waldrop scale score of 4.25 in a population of autistic children.[14]

Trixler’s group focused on 56 informative variants in schizophrenic and alcohol-dependent patients and made a distinction between minor malformations and phenotypic variants. They found that schizophrenic patients had higher rates of both some minor malformations (furrowed tongue, multiple buccal frenula and hemangioma) as well as phenotypic variants (protruding auricle and large tongue).[17]

Thyroid dysfunctions are more common in children with Down syndrome than in normal children. From 15 to 20% of children with Down syndrome have hypothyroidism. While investigating children with global developmental delay, it is also vital not to miss conditions which may be exacerbating it or those conditions which are treatable e.g., hypothyroidism.[18] Stavrakaki recorded that 27% of individuals with intellectual disability had anxiety disorder.[19] The comorbidity with other psychiatric illness e.g., depression is common. In more severe cases the behavioral symptoms associated with anxiety can be reliably assessed.[20] Vitielli *et al*, and Bodfish *et al*, reported that compulsions were significantly associated with stereotypies and self-injurious behavior (SIB).[21,22] There are arguments for SIB, compulsions and stereotypies to be considered as atypical presentation of Obsessive Compulsive Disorder.[23] Gravestock suggested that 1-19% of adults with disabilities living in the community and 3-42% of those living in institutions have a diagnosable eating disorder.[24]

Individuals with Down syndrome have frequently been described as having charming personalities in accordance with a positive Down syndrome personality stereotype.[25] Older children and young adults with Down syndrome are described as primarily positive mood and predictable in their behavior but less active and persistent and more distractible than older children as well.[26] In separate studies Waldrop and Halverson *et al*, found evidence of possible congenital contributes to individual differences in impulse control. This evidence is based on finding that relatively uncontrolled, fast moving, hyperactive behavior is related to presence of certain MPA in young children.[5] Waldrop and Halverson have argued that stability in the number of MPA and not the specific category of MPA as a predictor for behavioral problems.[8] However, this is not true for all cases. For example, a higher incidence of anomalies of the mouth have been linked to psychosis in several studies as well as in schizophrenic patients.[26]

The increased prevalence of MPA in the hand region of patients merit further interest since some groups of schizophrenic patients have shown aberrant dermatoglyphical patterns, which are also presumptive markers of prenatal neurodevelopmental disturbances.[14] About 60% of patients with schizophrenia have increased level of MPA (>6) in comparison to 5% of normal population.[16] The emotional problems in intellectually disabled persons are very severe and more pronounced than problems observed in general population. Over 500 recognized syndromes involving a genetic disorder have now been isolated and many have behavioral epiphenomena.[10] In two samples of 2-1/2-year children, the presence of multiple MPA was found to be associated with hyperkinetic, aggressive, impatient and intractable behaviors. Out of nearly 100 reliable behavioral variables, 18 in 1 sample and 16 in the other correlated with anomalies in the male subjects.[8]

## Materials and Methods

A careful dysmorphology exam is essential for the detection of MPA and because 71% of anomalies are present in the craniofacial area and the hands, careful attention to these areas can be helpful in diagnosing occult major anomalies.[11] The orofacial structures that do not form properly can result in problems with communication, emotional expression, mastication and deglutition. Anomalies that occur in the mouth can also lead to sucking problems and feeding irregularities during the first years of life that could also affect the mother-child relationship.[27] The behavioral phenotype is relatively a new concept. In a broad sense it seems to be a constellation of specific behaviors and specific disorder of genetic etiology.[9] An estimate that mental health problem is five times higher in intellectually disabled person is a conservative one.[28] Both in Western literature and in Indian context many studies have been done about the chromosomal abnormality of Down syndrome that is found by karyotyping, but most of them did not provide adequate information about its direct causal relationship with different phenotypic and behavioral variants. The aims and objectives of present study are:

To assess clinico-demographic profile of patients and subjects.

To find out MPAs and its incidence.

To study the behavioral profile among the people with intellectual disability.

The search for correlation between minor anomalies and behavioral profile.

To get additional information about type of chromosomal anomaly and correlation with intelligent quotient (IQ).

**Figure 1:**
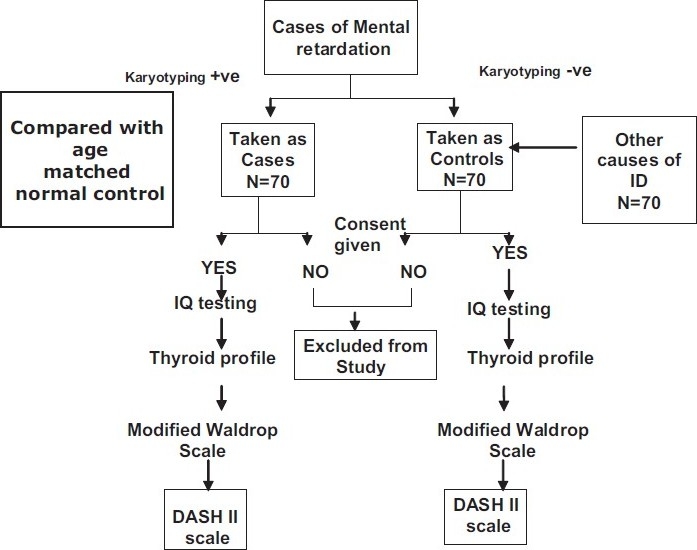
Algorithm of study design

The study design is a cross-sectional survey done independently by the three researchers. The study was conducted during the period from February 2007 to January 2009 [Figure 1]. The total sample size was 210 divided equally in three groups-cases (karyotype-confirmed Down syndrome group), control (other causes of intellectual disability) and normal (age-matched healthy population group). The MPA were assessed by using Modified Waldrop scale consisting of 18 plus 23 additional items.[8] The assessment of MPA takes only 15 minutes with very minimal removal of clothing (shoes and stockings). For practical reasons, visible surfaces like head, eyes, mouth, ears, hands and feet regions are studied. The items of diagnostic assessment scale for severely handicapped (DASH-II) scale comprises of a total of 83 items representing 13 disorder groups derived from subscales and individual items DSM-III R (American Psychiatric Association, 1987) as well as previously published studies of the population.[25] Three separate dimensions of behavior were used for rating namely frequency, severity and duration - for administrative convenience only frequency dimension had been analyzed. Each dimension provided for rating on one of three levels, scored 0, 1 or 2. The usual time required to apply this scale averages 60-90 minutes and less as the rater gets accustomed to use this scale.[20]

The inter-rater reliability of DASH-II mood subscale was found to be 0.88.[20] The depression subscale of the DASH-II displayed convergent validity of 0.75 with the Aberrant Behavior Checklist.[29]

## Results and Discussion

The three groups are represented uniformly as far as the population distribution is concerned. While age of Down syndrome cases ranged from 3 to 37 years that of comparison group 3 to 39 years and that of normal control group, 3 to 31 years. The sex difference in between the groups was not found to be statistically significant (χ^2^ = 5.833, *df* = 2, *P* = 0.054). The average paternal and maternal age at the time of childbirth in Down syndrome are 35.76 and 31.16 years, respectively, which corroborates the earlier findings that the incidence of Down syndrome increases with both increase in paternal and maternal age [Table 1]. The maternal age of marriage and income are other significant demographic variables (*P*<0.001). The only mosaic variety of Down syndrome in this study had total IQ of 64, with lower maternal age of childbirth (25 years) and less behavioral abnormalities. About 22.84% patients with Down syndrome also had hypothyroidism, and the only translocation variety case also had relatively higher total IQ (62) and borne by relatively younger mother (26 years). As the study areas are situated in the urban region most of the cases had come from the nuclear family with middle-class background with higher average parental age in comparison to the standard population. The Down syndrome group had significantly more MPA than other two groups and most of the MPA is situated in the global head region.

**Table 1:** Descriptive and ANOVA summary of demographical profile

Demographical variables	Group	N	Mean	SD	F	Significance (*P*-value)
Age	DS	70	16.30	10.288	1.771	0.173
	OTH	70	14.93	8.030		
	NOR	70	13.61	6.579		
Paternal age at childbirth	DS	70	35.76	7.264	11.731	< 0.001**
	OTH	70	32.51	4.886		
	NOR	70	31.50	3.446		
Maternal age at childbirth	DS	70	31.16	5.067	22.066	< 0.001**
	OTH	70	26.59	4.356		
	NOR	70	27.14	3.816		
Income	DS	70	3.06	1.020	3.724	0.026*
	OTH	70	2.90	0.837		
	NOR	70	3.29	0.617		
Maternal age of marriage	DS	70	28.60	4.308	74.373	< 0.001**
	OTH	70	22.64	3.750		
	NOR	70	22.09	2.048		
Birth order	DS	70	2.11	2.004	3.010	0.051
	OTH	70	1.57	0.894		
	NOR	70	1.66	1.062		

* *P* < 0.05

** *P* < 0.01;

DS indicates, Down syndrome; OTH, other causes of intellectual disability; NOR, normal.

The total score of Modified Waldrop scale (Ismail *et al*,) shows [Table 2] significantly higher values in the Down syndrome group (mean=17.04; SD= 5.462) than in the other intellectual disability group (mean=5.93; SD=2.628) and intellectually average age-matched group (mean=1.59; SD=1.378). Among the 13 major subscales in DASH II scale, the stereotypy (n=44, 62.9%), impulse control (n=36, 51.4%) and mania subscales (n=31, 44.3%) are present in significantly higher frequencies in the Down syndrome group, whereas organic subscale (n=48, 68.6%) and impulse control disorder subscales (n=42, 60.0%) more commonly present in the control group. [Table 3] The least frequent anomalies are schizophrenia and anxiety disorders in the Down syndrome group. Overall the behavioral abnormalities as evident from the scoring in the DASH II scale are more common in other non-Down syndrome cases of intellectual disability group than in Down syndrome group. The correlation matrix shows strong association (*P*<0.001) between the various grouped items of Modified Waldrop scale (including the anomalies in feet region) in cases of Down syndrome [Table 4] The correlation matrix between MPA and behavioral problems shows that not only anomalies around the mouth but also MPA distribution in other areas are also associated with behavioral abnormalities. Depression subscale is correlated with anomalies in the hands (*P*<0.001), feet and Waldrop total items (*P*<0.005). Mania item of DASH II scale is related with anomalies around the eyes (*P*<0.001), global head, mouth and Waldrop total score (*P*<0.005). SIB and total Waldrop score is negatively correlated with global head but positively related with anomalies around the ears. The eating disorder subscale is positively correlated to anomalies around the eyes and ears and the same correlation is seen between the sleeping disorder anomalies around the eyes (*P*<0.005). The items of DASH II scale that are not related to Waldrop scale are pervasive developmental disorder, schizophrenia, stereotypy, elimination, sexual, organic and impulse control disorder subscales [Table 5].

**Table 2:** Descriptive summary of modified Waldrop score in three groups

Group	N	Mean	Med	Min	Max	Sd
DS	70	17.04	18.0	6.0	30.0	5.462
Other ID	70	5.93	6.0	1.0	13.0	2.628
Normal	70	1.59	1.5	0.0	5.0	1.378

DS indicates, Down syndrome; NOR, normal.

**Table 3:** Frequencies of DASH II subscales in the three groups studied

Item	DS		Control		Normal	
	N	%	N	%	N	%
Anxiety	9	12.9	2	2.9	0	0.0
Depression	17	24.3	8	11.4	3	4.3
Mania	31	44.3*	7	10.0	2	2.9
PDD	28	40.0	23	32.9	0	0.0
Schizophrenia	5	7.1	3	4.3	0	0.0
Stereotypy	44	62.9*	41	58.6	1	1.4
Sib	6	8.6	28	40.0	0	0.0
Elimination	15	21.4	16	22.9	1	1.4
Eating	18	25.7	16	22.9	2	2.9
Sleep	20	28.6	18	25.7	3	4.3
Sexual	15	25.7	18	25.7	0	0.0
Organic	26	37.1	48	68.6*	0	0.0
Impulse	36	51.4*	42	60.0*	7	10.0

* Highest frequencies observed in the group;

DS indicates Down syndrome cases; PDD, pervasive developmental disorder; SIB, self-injurious behavior.

**Table 4:** Correlation matrix of grouped regions in Ismail 41 scale

Correlation	Global head	Eyes	Ears	Mouth	Hands	Feet	Waldrop total
Global head	1.000	0.642	0.743	0.771	0.655	0.655	0.878
Eyes	0.642	1.000	0.623	0.482	0.447	0.424	0.707
Ears	0.743	0.623	1.000	0.644	0.618	0.728	0.826
Mouth	0.771	0.482	0.644	1.000	0.577	0.562	0.836
Hands	0.655	0.447	0.618	0.577	1.000	0.698	0.754
Feet	0.655	0.424	0.728	0.562	0.698	1.000	0.777

In all cases *P* - value is <0.001

**Table 5:** Correlation between Ismail score and DASH-II score

	Global Head	Eyes	Ears	Mouth	Hands	Feet	Waldrop Total
ANX Pearson Correlation	0.170	-0.112	0.155	0.176	0.096	0.097	0.127
Sig (2-tailed)	0.160	0.356	0.200	0.145	0.430	0.423	0.293
N	70	70	70	70	70	70	70
DEPR Pearson Correlation	0.168	0.141	0.181	0.133	0.381**	0.252*	0.273*
Sig (2-tailed)	0.165	0.246	0.133	0.271	0.001	0.035	0.022
N	70	70	70	70	70	70	70
MANIA Pearson Correlation	0.253*	0.329**	0.231	0.275*	0.020	0.082	0.257*
Sig (2-tailed)	0.034	0.005	0.055	0.021	0.869	0.501	0.032
N	70	70	70	70	70	70	70
PDD Pearson Correlation	-0.159	-0.063	-0.115	-0.214	-0.179	-0.062	-0.206
Sig (2-tailed)	0.187	0.606	0.341	0.075	0.139	0.610	0.087
N	70	70	70	70	70	70	70
SCHZ Pearson Correlation	0.025	-0.071	0.021	0.131	-0.105	-0.133	0.000
Sig (2-tailed)	0.837	0.558	0.862	0.279	0.385	0.271	1.000
N	70	70	70	70	70	70	70
STEREO Pearson Correlation	0.102	0.126	-0.040	0.092	-0.030	-0.023	0.041
Sig (2-tailed)	0.401	0.299	0.740	0.450	0.803	0.850	0.733
N	70	70	70	70	70	70	70
SIB Pearson Correlation	0.285*	-0.203	-0.248*	-0.219	-0.228	-0.210	-0.291*
Sig (2-tailed)	0.017	0.092	0.038	0.068	0.058	0.080	0.015
N	70	70	70	70	70	70	70
ELIMIN Pearson Correlation	0.030	-0.058	0.088	0.130	-0.066	-0.032	0.047
Sig (2-tailed)	0.806	0.636	0.467	0.283	0.585	0.795	0.698
N	70	70	70	70	70	70	70
EATING Pearson Correlation	0.147	0.277*	0.264*	0.192	0.234	0.145	0.202
Sig (2-tailed)	0.225	0.020	0.027	0.111	0.051	0.230	0.093
N	70	70	70	70	70	70	70
SLEEP Pearson Correlation	0.118	0.301*	0.002	0.140	0.000	-0.074	0.088
Sig (2-tailed)	0.332	0.011	0.985	0.249	0.997	0.541	0.469
N	70	70	70	70	70	70	70
SEXUAL Pearson Correlation	-0.075	-0.051	-0.141	-0.007	-0.115	-0.072	-0.121
Sig (2-tailed)	0.538	0.673	0.245	0.953	0.341	0.552	0.319
N	70	70	70	70	70	70	70
ORG Pearson Correlation	-0.150	-0.067	-0.180	-0.174	-0.150	-0.004	-0.160
Sig (2-tailed)	0.210	0.584	0.135	0.150	0.216	0.972	0.186
N	70	70	70	70	70	70	70
IMP Pearson Correlation	0.012	0.125	0.066	0.037	0.070	0.067	0.084
Sig (2-tailed)	0.918	0.304	0.587	0.763	0.565	0.584	0.487
N	70	70	70	70	70	70	70

* Correlation is significant at the 0.05 level (2- tailed);

** Correlation is significant at the 0.01 level (2- tailed);

ANX indicates anxiety; DEPR, depression; PDD, pervasive developmental disorder; SIB, self-injurious behavior, SCHZ, schizophrenia; STEREO, stereotypy; ELIMIN, elimination; ORG, organic; IMP, impulse.

## Conclusion

The sample size is only modest (70 in each group). The increased sample size could have increased the power of tests. No follow-up studies have been done. The study design is the single observation cross-sectional survey, but behavioral features can vary with time. Some of the physical features are very rare especially in Asian countries. A better scale with wider applicability, reliability and validity is required to detect MPA in different ethnic background. There was always the possibility that some of the informants (the legal guardians) would provide inaccurate information especially while assessing the behavioral abnormalities of the subjects and the interpretation may be erroneous. To minimize this error, appropriate consultation has been taken with the senior faculties of the department as well as liaison services have been sought for from the Department of Pediatrics and retrospective patient record sheet has also been reviewed. The metabolic screening has not been done due to administrative inconvenience. Therefore, associated disorders of Inborn Error of Metabolism if present can not be excluded. The congenital CNS structural anomaly can not be screened out as the routine structural imaging has not been performed due to cost constraints.

Despite the limitations as mentioned above, the study generated valuable information as discussed and the authors believe that the results of this study will help further research work regarding the incidence of MPA and correlation of MPA with behavioral abnormalities in the Down syndrome population group. Not only the Down syndrome group, but also other causes of intellectual disability group needs to be worked out. The large scale study is required to see whether MPA can be a predictor of future behavioral characteristics which can have preventive, therapeutic, rehabilitative and prognostic implications.

Researchers believe that when a high number of MPAs as determined by the Waldrop scale are present, there are implications for behavioral variations. Recently, the literature is focused on the schizophrenic population. Although this study did not confirm the usefulness of screening the general population for MPAs as a predictor for behavior, the results demonstrate that the Down syndrome group has significantly more MPAs and a pattern of correlation between MPA and behavioral abnormalities in a large sample can be really worthwhile.
